# 3D Tissue and Organ Printing—Hope and Reality

**DOI:** 10.1002/advs.202003751

**Published:** 2021-03-11

**Authors:** Assaf Shapira, Tal Dvir

**Affiliations:** ^1^ Shmunis School of Biomedicine and Cancer Research Faculty of Life Sciences Tel Aviv University Tel Aviv 6997801 Israel; ^2^ Department of Materials Science and Engineering Faculty of Engineering Tel Aviv University Tel Aviv 6997801 Israel; ^3^ The Center for Nanoscience and Nanotechnology Tel Aviv University Tel Aviv 6997801 Israel; ^4^ Sagol Center for Regenerative Biotechnology Tel Aviv University Tel Aviv 6997801 Israel

**Keywords:** 3D printing, bioinks, biomaterials, cells, tissue engineering

## Abstract

Three‐dimensional (3D) bioprinting is an emerging, groundbreaking strategy in tissue engineering, allowing the fabrication of living constructs with an unprecedented degree of complexity and accuracy. While this technique greatly facilitates the structuring of native tissue‐like architectures, many challenges still remain to be faced. In this review, the fruits of recent research that demonstrate how advanced bioprinting technologies, together with inspiring creativity, can be used to address these challenges are presented and discussed. Next, the future of the field is discussed, in terms of expected developments, as well as possible directions toward the realization of the vision of fully functional, engineered tissues, and organs. Last, a few hypothetical scenarios for the role 3D bioprinting may play in future tissue engineering are depicted, with an emphasis on its impact on tomorrow's regenerative medicine.

## Graphical abstract

3D bioprinting is an emerging strategy in tissue engineering. While it greatly facilitates the structuring of tissue‐like architectures, many challenges still remain. Here, the fruits of the latest research that address these challenges are presented. The future of the field is then discussed and hypothetical scenarios for the role of 3D bioprinting in future tissue engineering and regenerative medicine are depicted.

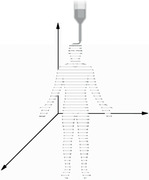

## 1 Introduction

Since ancient times, humans have been fascinated by the unimaginable complexity of living creatures. The orchestrated function of multiple structures with incredible geometries ignited the imagination of our ancestors, making them raise existential questions. The invention of the microscope further enhanced this enthusiasm, revealing the existence of a new, concealed world of sophisticated, functional, tiny bio‐architectures. For medical experts and clinicians, however, these observations were accepted with an ambivalent feeling. On the one hand, they shed light on the mechanisms that support life with far‐reaching implications on medical care. On the other hand, they stressed the difficulties one may face while trying to regenerate such complicated, delicate systems. Nevertheless, the idea to artificially construct living tissues, or even whole organs, has never been abandoned, setting up the base for the rising field of tissue engineering (TE). The concept of TE is generally focused on the construction of acellular or cellularized patches that can be implanted alongside or instead of a damaged tissue, leading to regeneration of its hampered or lost function. To achieve an optimal therapeutic effect, the engineered patch is usually designed to mimic the native tissue in terms of the cellular, biochemical, mechanical, and structural features.^[^ ^1^, ^2^ ^]^ While numerous studies have demonstrated the feasibility of this concept, in the vast majority of these cases the structure of the engineered tissues is still considerably different from that of their native counterparts. This can be largely attributed to the fact that traditional fabrication methods do not provide an adequate capacity to precisely control the spatial positioning of the building materials. Moreover, while some forms of basic biostructures can be generated by spontaneous cellular organization processes, these are very difficult to control and manipulate. Given the high compositional and structural complexity of living tissues, a fabrication method capable of precisely depositing different materials and cells in pre‐defined locations in the 3D space is highly desirable. This capacity was introduced with the development of techniques for additive manufacturing (AM), commonly known as “3D printing.”^[^ ^3^, ^4^ ^]^

The “classic” AM/3D printing process can be described as a procedure in which a 3D physical object is built, layer‐by‐layer, on the basis of data from a computer‐aided design (CAD).^[^ ^4^, ^5^, ^6^ ^]^ Traditionally used for the rapid prototyping of objects made of industrial‐grade plastic, glass, metal, ceramics, etc., the technique has recently been adopted and modified for the fabrication of both acellular and cell‐containing tissue‐like structures made of biocompatible, cell‐friendly materials. This technology, termed “3D bioprinting,” has revolutionized the field of TE by taking it a step forward toward a new era in which the fabrication of complex, composite bio‐architectures is within reach.^[^ ^7^ ^]^ Indeed, the last years have been characterized by a massive burst of intriguing research and fascinating developments in this field. 3D bioprinting techniques have been fine‐tuned and refined so that they can now be used to deposit a growing diversity of meticulously formulated biomaterials with unprecedented accuracy, without compromising on the viability of encapsulated cells.^[^ ^8^, ^9^, ^10^, ^11^, ^12^ ^]^ The fact that 3D biofabricated tissue‐like structures share more and more features with their natural equivalents indicates the enormous potential of the technology to bring us closer to the desired goal of manufacturing functional replacement body parts. Nevertheless, there are still many challenges to overcome, some of which relate to the printing technology itself, some to the structural and supporting biomaterials, and some derive from the quality of the biostructures to be printed. In this article, we briefly discuss several of the prominent, recently published works in which innovative approaches and advanced technologies were harnessed to face some of these challenges. An emphasis is given to extrusion and photopolymerization‐based fabrication strategies that allow structuring with an exceptional degree of complexity and accuracy.^[^ ^12^, ^13^ ^]^ After a short review of the state of the art, we bring our own insights and vision for the near‐ and far‐future of 3D bioprinting and its foreseen impact on research and clinical practice.

## 2 Facing the Challenges

3D bioprinting techniques are based on similar principles to conventional AM approaches, for example, extrusion, inkjet, and light‐based printing (which includes stereolithography (SLA), two‐photon polymerization (2PP), and laser‐assisted printing (LAP)). These techniques, however, have undergone modifications and adaptations dictated by the nature of the building materials, incorporated cells, and working environment.^[^ ^9^ ^]^ That is to say that the processes should be gentle enough so as not to involve any steps that expose delicate printing materials and loaded biofactors to conditions that may adversely affect their quality. Things get much more complicated, though, when living cells are present in the formulation (referred to as a “bioink”^[^ ^14^ ^]^). In these cases, the process becomes even less forgiving, forcing the user to work in a very narrow range of conditions. Last, to all of these restraints is joined the challenge of performing the process under sterile conditions. While bioprinting processes are, by far, less permissive than the more common, conventional AM techniques, they are not less capable of endowing the user with extraordinary creative liberty. To realize this power and bring it into practice, however, one should take advantage of the unique capabilities of the specific working platform, while at the same time confronting its challenges. By integrating biology with excellent engineering, leading research groups have creatively used advanced, customized 3D bioprinting techniques to define the cutting edge of engineered tissues and biostructures. We have categorized these recent works according to the way they addressed three main challenges in the field: the complexity of the fabricated structure, the accuracy of the printing, and the speed of the process.

### 2.1 Making It Complex

**Figure 1:**
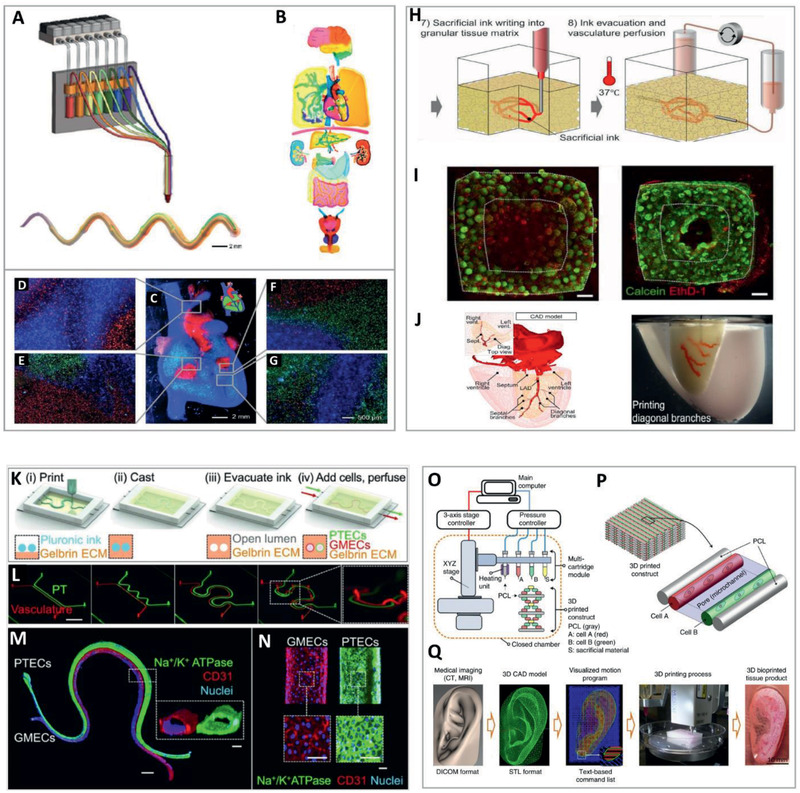
Printing of complex structures. Continuous multimaterial extrusion bioprinter. A) Schematic illustration of the mutimaterial printhead and a photograph of a printed microfiber. B) Human organ‐like structures bioprinted using multiple bioinks. Lower panel: C) A macroscopic image of a multicomponent heart‐like structure loaded with fluorescent microbeads and D–G) microscopic images of junction regions showing coexistence of differently pre‐labeled embedded cells. Adapted with permission.^[^ ^21^ ^]^ Copyright 2016, Wiley‐VCH. Sacrificial writing into functional tissue (SWIFT). H) Process illustration. I) viability staining showing improved cell survival in channeled, perfused tissue (right) versus non‐channeled tissue (left). Scale bars: 500 µm. J) The left anterior descending (LAD) artery together with diagonal and septal branches were printed into septal‐anterior wall wedge of cardiac tissue matrix (right), with structural data derived from a 3D CAD model downloaded from the NIH 3D Print Exchange (left). Adapted with permission.^[^ ^29^ ^]^ Copyright 2019, AAAS. A 3D printed vascularized proximal tubule model. K) Model design. L) Printing of several model architectures with an increasing degree of complexity (Scale bar: 10 mm). M,N) Immunofluorescence staining of a cellularized printed tissue stained for Na+/K+ ATPase (Green, in proximal tubule lined with epithelial cells), CD31 (Red, in vascular channel lined with endothelial cells) and nuclei (Blue). Scale bars: 1 mm in (M), 100 µm in inset, and in (N). Reproduced with permission.^[^ ^31^ ^]^ Copyright 2019, National Academy of Sciences. Biofabrication of mechanically stable, human‐scale tissue constructs using integrated tissue‐organ printer (ITOP). O) Illustration of the ITOP system designed to deliver multiple cell‐laden hydrogels, supporting PCL and sacrificial Pluronic‐F127 and P) the basic patterning of a printed 3D architecture. Q) A representative 3D bioprinting process from the data acquisition stage to a fabricated, engineered tissue product. Reproduced with permission.^[^ ^32^ ^]^ Copyright 2016, Springer Nature.

Most of the work published during the earliest years of TE was based on the fabrication of homogenous, porous scaffolds with simple geometries. These scaffolds were either acellular or contained unpatterned cells.^[^ ^15^ ^]^ While this was acceptable at the time as a proof of concept, the design of modern engineered bio‐constructs has evolved to better reflect the complex composition and architecture of native tissues.^[^ ^16^ ^]^ A special emphasis was given to the multiplicity of biomolecules and cell types, the spatial arrangement of which is crucial for proper physiological function. An intuitive example in this regard is the human skin, where the proper function depends on a particular arrangement of distinct layers, each dominated by a specific type of cells.^[^ ^17^, ^18^, ^19^ ^]^ Recent advances in mechanical and material engineering have led to the accelerated development of extrusion‐based 3D bioprinters. These can be loaded with a wide variety of materials and cells, which, when forced out through a printhead nozzle, form a continuous strand.^[^ ^13^, ^20^ ^]^ When precisely deposited in pre‐defined positions according to a meticulously planned digital design, heterogeneous, composite, tissue‐like structures can be fabricated.^[^ ^8^ ^]^ An example of a unique method for fabricating such structures has been presented by Liu et al.^[^ ^21^ ^]^ In this study, the authors developed a 3D bioprinter capable of fabricating structures with high compositional complexity using a single printhead. The printer, which consisted of a bundle of seven thin capillaries individually connected to unique bioink reservoirs, enabled the extrusion of multiple bioinks in a fast and continuous manner. In an impressive eye‐catching demonstration, cellular and acellular, sophisticated, planar, and 3D patterns were printed using both individual and simultaneous bioink injection modes (**Figure** **1**A–G). Importantly, the constructs were fabricated at a speed that is up to 15 times faster than that which is achieved when printing using existing nozzle‐based platforms without compromising either accuracy or cell viability. Shape fidelity was degraded to some extent, though, as a result of partial collapse of large multi‐layered structures. With printing resolution of 100–200 µm and the ability to generate gradient structures that mimic those occurring in natural tissues, this bioprinting strategy is definitely an interesting choice for complex, multimaterial 3D structuring.^[^ ^21^ ^]^

In addition to material and cell heterogeneity, another basic feature of higher organisms is the presence of a vascular system that ensures a constant supply of oxygen and nutrients and removal of waste from each and every cell in the body. As a requirement for the survival of cells in 3D structures, where the rate of diffusive transport into the core of the bulk is insufficient, vascularization has become a major aim for tissue engineers.^[^ ^22^ ^]^ Endothelial cells, seeded in engineered tissues, can spontaneously organize into vessel‐like structures that are able to anastomize with the host. Nevertheless, this process is relatively slow and cannot keep pace with the metabolic requirements of newly implanted tissue.^[^ ^23^ ^]^ For this reason, the strategy of generating pre‐vascularized engineered tissues that can be rapidly perfused upon completion of the fabrication process has gained popularity. The last decade has been characterized by an abundance of publications in which different concepts were applied to accomplish this goal.^[^ ^22^, ^24^, ^25^, ^26^ ^]^ One common fabrication strategy is to use fugitive/sacrificial materials, such as Pluronic F127, gelatin, and carbohydrates, that temporarily define and support the structure of the printed vessel network within the engineered, surrounding parenchyma. Upon completion of the fabrication process, the structure is cured while the sacrificial material is discarded. This process generates voids that can be perfused with oxygen and nutrient‐rich cell‐media throughout the whole volume of the construct.^[^ ^27^, ^28^ ^]^ A distinguished work that elegantly demonstrated such a strategy was recently published by Lewis and co‐workers.^[^ ^29^ ^]^ In this work, the authors developed a biomanufacturing method referred to as “SWIFT” (sacrificial writing into functional tissue). At the core of this strategy, induced pluripotent stem cell (iPSC)‐derived organoids are grown and harvested to generate organ‐specific building blocks. These are then mixed with extracellular matrix (ECM) solution and compacted to yield a densely cellular, granular matrix. Next, a gelatin‐based sacrificial ink is deposited into the matrix, which embraces and stabilizes the printed pattern by virtue of its self‐healing, viscoplastic properties. Curing the matrix by incubating at 37 °C and removing the liquefied, embedded, fugitive ink then yields a channel system within the living construct. The resulting channels can then be perfused with endothelial cells that cover the inner part and form a monolayer on the lumen, recapitulating blood vessel endothelium. The researchers showed that SWIFT‐printed perfused vascularized structures resulted in a significant improvement in cell viability compared to non‐vascularized controls. As expected, the most dramatic effect was observed at the core of the constructs. The SWIFT method was then used to demonstrate the fabrication of a perfusable, engineered cardiac tissue that remained viable and beat synchronously over a 7‐day period^[^ ^29^ ^]^ (Figure 1H–J). A second publication from this group gave yet another example of mimicking the complex architecture of native tissue. This time, the researchers focused on modeling the proximal tubule (PT) of the kidney. By using Pluronic F127 as a fugitive ink, a PT model was fabricated, consisting of an ECM‐embedded, open lumen circumscribed by PT epithelial cells (PTECs). A perfusable tissue chip was used to house the model, providing it with physiological shear stresses. As demonstrated, the resulting 3D PTs promoted the formation of a renal tubular‐like epithelium. This cell monolayer exhibited several morphological features and functional markers akin to native PTECs, including the presence of cilia, albumin uptake, and the expression of Na^+^/K^+^ ATPase, Aquaporin 1, and K cadherin.^[^ ^30^ ^]^ In a follow‐up study, the researchers enhanced the model to also contain a second, adjacent, endothelialized open lumen that recapitulated a peritubular capillary (Figure 1K–N). The dually perfused construct enabled the investigation of selective reabsorption of solutes via tubular–vascular exchange, akin to the native kidney tissue. This physiological‐like behavior indicates the capacity of the platform to serve as a model to study kidney function under both homeostasis and disease conditions.^[^ ^31^ ^]^ It should be noted, however, that in the three aforementioned works, the printed fugitive ink is embedded in casted media that eventually becomes an integral part of the final construct. This may limit the construct's design, as the printer is unable to control either the composition of this component, or its geometry, which is dictated by the shape of the cast mold. In addition, a second step, post‐printing perfusion, needs to be introduced into the fabrication scheme in order to obtain cell‐lined channels.

**Figure 2:**
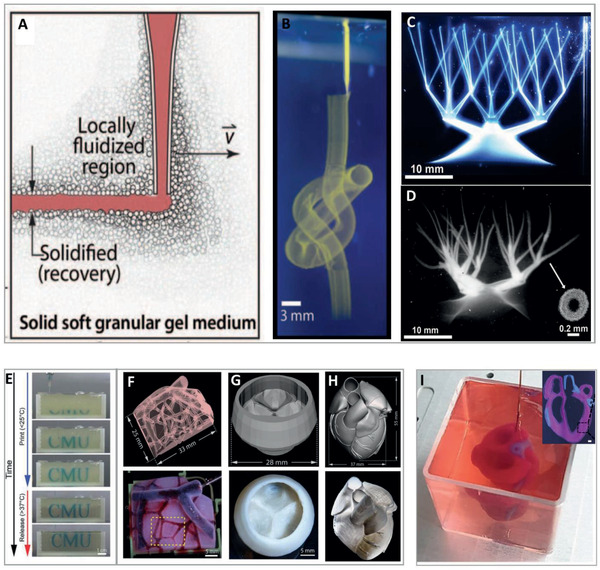
Printing of complex structures (continued). Writing inside Carbopol microgel support bath. A) Schematic representation of the principle behind printing inside a granular support medium. B) Printing of complex structures by extrusion of fluorescent microsphere suspension inside a microgel support bath. C) A continuous network of hollow vessels made of photo‐crosslinkable PVA before and D) after crosslinking and extraction from the support. Adapted with permission.^[^ ^33^ ^]^ Copyright 2015, Published by AAAS. 3D bioprinting using freeform reversible embedding of suspended hydrogels (FRESH). E) Time‐laps sequence of printing using FRESH. F) Perfused 3D vascular network, G) tri‐leaflet heart valve and H) neonatal‐scale human heart printed from acidified collagen. The underlying digital models are shown above the pictures of the actual printed constructs. Adapted with permission.^[^ ^35^ ^]^ Copyright 2019, AAAS. I) 3D bioprinting using pepsinized ECM‐based bioinks in particulate, alginate‐xanthan gum hybrid support media. The main panel shows an in‐process image of a printed, small‐scale cellularized human heart with major blood vessels fabricated using two bioinks. Reproduced under the terms of the CC‐BY license.^[^ ^38^ ^]^ Copyright 2019, the Authors, Published by Wiley‐VCH. Inset: A printed, acellular coronal cross‐section of the miniaturized heart. The structures were supplemented with colored microbeads for visualization. Scale bar: 1 mm. Adapted with permission.^[^ ^37^ ^]^ Copyright 2020, IOP.

Another layer of complexity that characterizes the tissues and organs of higher organisms is their geometry and macro‐structure. This constitutes a significant hurdle, especially for the printing of large, volumetric structures, as many materials commonly used in bioprinting are soft. The weak mechanical properties of these materials are incapable of providing adequate self‐support, at least until the constructs are fully cured. This typically results in a distorted geometry of multi‐layered constructs that may eventually collapse under their own weight. A similar problem also exists when the geometry of the structure dictates the printing of bridges (when a material is deposited on “thin air” without an underlying material layer) and/or overhangs (when an underlying material layer provides only partial support). To address this problem, several strategies have been implemented, most of which are based on the integration of some sort of permanent or temporal support for the printed structures.^[^ ^28^ ^]^ A comprehensive work performed by Kang et al. provided an excellent example of such a strategy.^[^ ^32^ ^]^ In this work, Pluronic F127 and poly(*ε*‐caprolactone) (PCL) were used as temporal and permanent printing materials, respectively, to support the fabrication of cellular, human‐scale, tissue constructs. These materials were loaded, alongside cell‐laden composite hydrogels, into a multifunctional system denoted as an “integrated tissue‐organ printer” (ITOP). The device, equipped with multiple extrusion‐based cartridges, was used to fabricate porous, volumetric biostructures on the basis of digital data acquired by medical imaging modalities (Figure 1O–Q). Externally supported by the fugitive Pluronic F127 and internally by PCL, structurally stable constructs of a mandible and a calvarial bone, as well as ear cartilage and skeletal muscle, were fabricated. The viability of cells inside these constructs was maintained with a constant increase in cell number over a 15‐day period. Importantly, in vivo structural robustness, host integration and tissue formation were well evident in animal‐implantation experiments.^[^ ^32^ ^]^ Another approach to support the biofabrication of volumetric structures composed of soft materials has been proposed by Bhattacharjee et al. and Hinton et al.^[^ ^33^, ^34^ ^]^ In two innovative works, the authors demonstrated a technique in which free‐form 3D printing is performed inside non‐thixotropic, particulate gel. This is achieved by virtue of the capacity of the granular material to fluidize around the traversing writing needle and at the point of injection, while rapidly solidifying to embed the extruded material behind the moving tip (**Figure** **2**A). The transparent, granular support medium that was developed by Bhattacharjee et al. was composed of jammed, hydrogel micro‐particles made of Carbopol, a cross‐linked polyacrylic acid copolymer. Extrusion of a wide variety of soft materials into this medium enabled the fabrication of complex, hierarchical structures with features ≈100 µm in diameter (Figure 2B–D). Moreover, living cells could be deposited and grown inside the particulate support material when prepared using growth medium as a solvent. The printed construct, which was embraced and stabilized by the support medium throughout the whole fabrication process, could be cured during or after the writing. As Carbopol cannot be liquefied or degraded by gentle, cell‐friendly treatments, extraction of the printout was performed by washing.^[^ ^33^ ^]^ It should be taken into account, however, that this mechanical extraction step may jeopardize the integrity of delicate structures. Moreover, removal of the support from narrow or internal voids could be very challenging.

Circumventing this difficulty, Hinton and colleagues introduced a process termed “freeform reversible embedding of suspended hydrogels” or “FRESH.” In this technique, a semi‐transparent support medium, composed of gelatin microparticle slurry, embraces the extruded material and preserves the geometry of the plotted shape. The printed construct, which undergoes curing concurrently with and/or after the completion of the writing process, can then be easily extracted by melting the granular gelatin support at a cell‐friendly temperature of 37 °C. Using the FRESH method for printing natural biopolymers, the researchers demonstrated the fabrication of complex acellular bio‐architectures such as a femur, a coronary arterial tree, a heart, and a brain.^[^ ^34^ ^]^ In a follow‐up study, the group proved the capability of the system to support the printing of acellular heart components, ranging in scale from capillaries to a tri‐leaflet valve and finally to a full organ (Figure 2E–H). This was performed using acid‐solubilized, high‐concentration collagen ink that cured while undergoing rapid neutralization upon extrusion into the granular gelatin support. This rapid equilibration to physiological pH was also found to allow for cells to be safely deposited, in a second step, in close proximity to the collagen component. Using a dual‐material printing process with collagen ink as the structural component and a high cell‐density bio‐ink, a contracting, cellular model of a heart's left ventricle was fabricated.^[^ ^35^ ^]^ The FRESH technique also served as the means for the fabrication of a synchronously contracting human chambered muscle pump. This time, a photo‐crosslinkable ECM formulation containing human iPSCs (hiPSCs) was used as a bioink to print two‐chambered structures with a vessel inlet and outlet. The cells then expanded and differentiated into cardiomyocytes (CM) within the photo‐cured structure. This in situ proliferation and differentiation strategy resulted in enhanced cell density and tissue connectivity, manifested as contiguous electrical function and pump dynamics. Nevertheless, it should be noted that this approach does not allow for the generation of constructs containing more than a single cell type since all the cells in the printout are inevitably treated with the same differentiation protocol.^[^ ^36^ ^]^

It is worth noting that, while the ease of printout extraction is a major strength of the FRESH method, the mechanism behind it concurrently limits its application. That is to say, the heat‐sensitivity of the gelatin particles restricts the use of printing materials that require prolonged curing at elevated temperatures, such as the commonly used pepsin‐treated ECM‐derived collagen preparations. In contrast to acid‐solubilized collagen, pepsin‐treated ECM‐derived collagen remains soluble at cell‐friendly pH (and thus can be used to encapsulate living cells) and gradually undergoes physical crosslinking at body temperature.

Recently, our group presented a modified version of a support medium, specifically developed for the printing of cell‐containing, pepsin‐treated, neutral, ECM‐based bioinks. This transparent, hybrid formulation, comprised of calcium‐alginate nanoparticles and xanthan gum, is thermally stable. Thus, it allows the thermal curing of collagenous bioinks upon extended incubation at 37 °C. Extraction from the support, in this case, is performed by using a delicate treatment with alginate‐degrading enzymes, or, alternatively, by calcium chelation.^[^ ^37^ ^]^ It should be noted, however, that these extraction procedures require the addition of external reagents. In addition, they usually take longer to accomplish than the above‐mentioned FRESH technique. Taking advantage of the high printing accuracy that can be achieved using this hybrid support medium, we were able to fabricate complex multimaterial geometries and cellular anatomical‐like structures. As a demonstration, we fabricated miniaturized cellular human hearts containing the major blood vessels (Figure 2I). Importantly, in this study, both the cells and the ECM‐based component of the bioinks were derived from a single human omentum tissue.^[^ ^38^ ^]^ It should be stressed that these organ‐like structures lacked internal branched vascular networks and were not tested for electromechanical function. Nevertheless, the presented capability, although still far from realization in the clinic, represents a significant step toward the 3D printing of fully personalized tissues and organs.

Overall, the described studies demonstrate the potential of innovative extrusion‐based bioprinting strategies to fabricate constructs with an exceptional degree of complexity. This potential can be attributed to the ability of these methods to accurately deliver a diversity of materials and cells to pre‐determined spatial positions, whether on top of a substrate or within a surrounding medium. However, this versatile scheme has some points of weakness stemming from the mechanism of dispensing materials through a nozzle. The first is the limited resolution that can be achieved. As a rule, a higher resolution requires the use of a finer dispensing nozzle. Unfortunately, narrowing the nozzle through which the materials pass results in the application of increased shear forces that may eventually rupture the encapsulated cells. This restricts the extrusion of bioinks to nozzles with an inner diameter of ≈150 µm, thus limiting the printing resolution of cellular constructs to approximately this value.^[^ ^13^, ^39^ ^]^ The second limitation relates to the process throughput, the effect of which is most pronounced when fabricating large objects. This limitation results from the localized nature of the material deposition mechanism, which relies on movements of the printhead and/or printer's stage for plotting the pattern of each layer.^[^ ^13^ ^]^

The weaknesses of this printing strategy, as well as of other fabrication schemes that will be outlined below, may be addressed by creative means and new concepts. These are discussed in the “Future Perspectives” section below.

### 2.2 Making It Accurate

**Figure 3:**
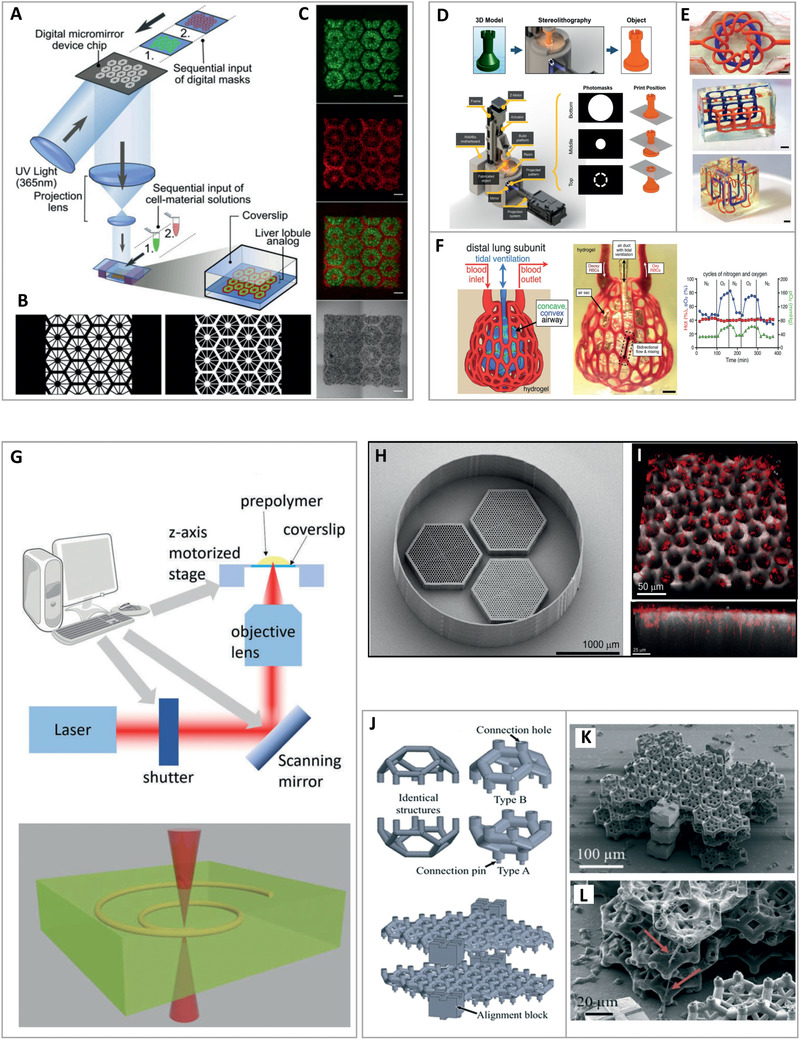
High‐accuracy printing. 3D bioprinted hepatic construct. A) Illustration of the two‐step, projection‐based stereolithography approach in which B) sequential exposure to two complementary shapes of patterned UV light resulted in C) liver lobule‐like structures containing hepatic cells (green) and supporting cells (red). Scale bars: 500 µm. Reproduced with permission.^[^ ^43^ ^]^ Copyright 2016, PNAS. Fabrication of complex, vascular architectures in biocompatible hydrogels. D) Schematic representation of a 3D printing process based on projection stereolithography. E) Perfused, entangled vascular networks printed within hydrogels. Scale bars: 3 mm. F) A scheme of a distal lung subunit (left), an actual printed structure during red‐blood cells (RBCs) perfusion and tidal ventilation (center), and a graph showing the RBC sensitivity to ventilation gas (right). Scale bar: 1 mm. Adapted with permission.^[^ ^45^ ^]^ Copyright 2019, AAAS. G) The two‐photon polymerization (2PP) fabrication method. A focused infrared or near‐infrared light is emitted from a femtosecond laser into a volume of photo‐crosslinkable substance to induce polymerization only at the focal point. Adapted with permission.^[^ ^46^ ^]^ Copyright 2018, Royal Society of Chemistry. 2PP‐fabricated retinal cell grafts. H) A scanning electron microscope image showing three scaffolds surrounded by a retaining wall. Each scaffold presents a different vertical pore size (25, 20, or 15 µm) and a horizontal pore size of 7 µm. I) A fluorescence image of a scaffold containing 25 µm vertical pores loaded with retinal progenitor cells (red). The bottom panel provides a side view, showing that the cells formed neuronal processes that extended into and aligned with the vertical pores. Adapted with permission.^[^ ^48^ ^]^ Copyright 2017, Elsevier. Generation of 3D cell networks using 2PP‐fabricated microcage‐containing scaffolds. J) The concept of micro‐scaffolds for confined cell growth. Blocks of complementary, half‐cell cages in the shape of truncated octahedrons are designed and printed. Cells are then seeded and grown inside the hemispherical containers, followed by stacking the cellular structures one on top of the other. K,L) Scanning electron microscopy image of a tri‐layer stack, with neurites projecting from the cages (red arrows) to establish connections between neighboring confined PC12 cells. Adapted with permission.^[^ ^50^ ^]^ Copyright 2019, The Royal Society of Chemistry.

Sophisticated geometries and micro/nano‐scale features are basic properties of biological structures. Obviously, as recapitulation of the native tissue architecture is fundamental for regenerative medicine, vast efforts have been invested in the development of accurate, ultra‐high‐resolution fabrication techniques. When it comes to sketching with high accuracy, a creator will tend to pick the finest writing implement that comes to hand. In the case of 3D printing, light is definitely the sharpest pencil in the box. SLA is a light‐assisted 3D printing method based on photopolymerization. In this method, a photo‐sensitive resin is successively cured, layer‐by‐layer, by either a point‐scanning laser beam (referred to as “direct/laser write” or “scanning SLA”) or selective exposure to a projected image plane (referred to as “projection‐based stereolithography,” PSL).^[^ ^12^, ^40^, ^41^, ^42^ ^]^ An inspiring demonstration of using SLA to create bio‐mimicking structures was provided by Chen and colleagues. In their research, the group generated a 3D hepatic model containing hiPSC‐derived hepatic progenitor cells cultured with supporting endothelial cells and adipose‐derived stem‐cells.^[^ ^43^ ^]^ To recapitulate the native liver module architecture, the researchers encapsulated the cells in photopolymerizable gelatin methacrylate (GelMA) and glycidal methacrylate‐hyaluronic acid (GMHA) hydrogels. These were then used as printing substances in a rapid, two‐step fabrication process, in which complementary shapes were generated by exposure to patterned UV light. The procedure resulted in constructs that consisted of microscale hexagonal lobule units of liver cells and supporting cells (**Figure** **3**A–C) that showed improved morphological organization and higher liver‐specific gene expression in comparison to two‐dimensional (2D) or hepatic progenitor cells‐only models. Moreover, the engineered tissues exhibited enhanced metabolic product secretion and induction of cytochrome P450, a family of key enzymes in liver drug metabolism.^[^ ^43^ ^]^ In a follow‐up study, the researchers used a similar printing technique to fabricate biomimetically patterned cellular heart and liver tissue constructs.^[^ ^44^ ^]^ In this work, the hydrogels used for cell encapsulation were based on photo‐crosslinkable decellularized‐ECM incorporating tissue‐specific, native biochemical constituents. These materials were shown to provide the encapsulated hiPSC‐derived cells with a highly supportive environment for maturation and organization. Importantly, this was done without compromising on design complexity and printing resolution, thus allowing the fabrication of structures with 30 µm features.^[^ ^44^ ^]^ Overall, these meticulously engineered tissues are definitely a step forward toward the development of improved, physiologically relevant in vitro models for disease studies, personalized medicine, and drug screening. It should be noted, though, that the above‐mentioned cellular constructs were not designed as thick, multilayered structures. Rather, they were built as low‐profile microarchitectures with a width and length of 3 mm and a thickness of only 250 µm. In other words, while the cells indeed experienced a true 3D environment, the macrostructure was more like that of a thin sheet.

A different approach for harnessing the power of SLA to accurately fabricate sophisticated geometries was presented by Grigoryan et al.^[^ ^45^ ^]^ In a colorful article, the researchers developed a modified PSL scheme capable of printing at a high resolution of 50 µm. The fabrication technique was initially utilized to produce poly(ethylene glycol) diacrylate (PEGDA) hydrogels containing intricate vascular architectures with functional internal topologies such as mixers and valves. Next, it served to explore the oxygenation and flow of human red blood cells (RBCs) during tidal ventilation. To this end, the authors developed a bioinspired alveolar model, in which RBCs were perfused through ensheathing vasculature that closely tracks the curvature of 3D airway topography. Tidal ventilation with oxygen caused a distention of the airway upon inflation, leading to the compression of adjacent blood vessels and the redirection of fluid streams to neighboring vessel segments. Furthermore, the perfused RBCs were found to respond to the ventilation. Expectedly, they presented significantly higher oxygen partial pressure and saturation relative to deoxygenated RBCs that were loaded at the inlet, or ventilated with nitrogen gas (Figure 3D–F). Next, the authors used their customized printing scheme to fabricate cellular structures made of PEGDA:GelMA‐based bioinks. To this end, lung‐mimetic architectures were populated with human lung fibroblasts in the bulk of the interstitial space, and human epithelial‐like cells were attached to the airway lumen. In another demonstration, human mesenchymal stem cells within fabricated hydrogels were found to maintain high viability for 24 h. The cells also showed osteogenic differentiation as a function of soluble factor delivery via vascular perfusion. Last, implantation experiments were performed in mice, demonstrating the in vivo survival and activity of engineered cellular hepatic tissues with an incorporated perfusable vasculature.^[^ ^45^ ^]^ The unprecedented degree of geometrical intricacy achieved by this rapid, precise, and cell‐friendly process, constitutes a significant milestone in the production of functional, vascularized, bio‐mimicking constructs. This advance may constitute the basis for the development of more accurate and physiologically relevant tissue models, accelerating progress in biomedical and pharmacological research. Limited compositional complexity, however, is still a major downside of this printing scheme, as will be elaborated further on.

While SLA is a preferred technique for printing accurate constructs at microscale resolution, it is by far the only strategy that is commonly used for the precise fabrication of sub‐micrometer features. This can be optimally achieved by virtue of a distinct type of laser‐based direct writing system: the highly precise two‐photon polymerization (TPP/2PP) method. In this method, characterized by a spatial resolution of down to 100 nm, a focused infrared or near‐infrared light is emitted from a femtosecond laser to induce polymerization inside a volume of photo‐crosslinkable substance. As the photon density required for polymerization is reached only at the focal point, a defined 3D structure can be patterned by moving the beam focus and/or the photo‐reactive material in the *X*, *Y*, and *Z* axes^[^ ^46^, ^47^ ^]^ (Figure 3G). Worthington et al. described a photoreceptor cell replacement concept for the treatment of retinal degenerative blindness using 2PP‐fabricated retinal cell grafts.^[^ ^48^ ^]^ The group used 2PP to recapitulate the fine natural structure of the outer retina, in which photoreceptor cells are tightly packed and aligned parallel to the light path. Using this precise fabrication method, non‐degradable 3D scaffolds with closely packed vertical pores 25 µm in diameter were fabricated. Interconnected, 7 µm horizontal pores were introduced to these 1 mm‐wide and 120 µm‐high structures in order to facilitate the diffusion of nutrients and oxygen. hiPSC‐derived retinal progenitor cells were then loaded into the scaffolds, forming neuronal processes that extended into and aligned with the vertical pores. Cell bodies were also found to populate the structure's columns, with the latter providing them with a proper vertical guidance^[^ ^48^ ^]^ (Figure 3H,I). The design of these constructs constituted the basis for a follow‐up study in which degradable, biocompatible, two‐photon polymerized PCL‐based scaffolds were fabricated. No inflammation, pyrogenicity, or other local or systemic toxicities were observed following sub‐retinal implantation of cell‐free scaffolds, indicating their future potential in the treatment of retinal degenerative diseases.^[^ ^49^ ^]^

The ultra‐high‐resolution capacity of 2PP has also been utilized for structuring stackable micro‐scaffolds comprised of synthetic photoresist. These scaffolds were engineered to allow confined cell growth in a specific, pre‐determined spatial organization. In these constructs, developed by Larramendy et al., blocks of complementary, half‐cell cages in the shape of truncated octahedrons were designed as stackable structural layers.^[^ ^50^ ^]^ Neuron‐like PC12 cells were then seeded and grown inside the hemispherical containers, followed by stacking the cellular structures one on top of the other. As the 50 µm‐diameter containers were designed as cages that restrain the cell bodies, cell‐to‐cell connections could only be realized between neurites. Indeed, neurites were found to project from the hexagonal openings of the cages and interact with those of neighboring cells, a first step toward the establishment of a 3D neuronal network. Such a technique thus holds potential for applications in which the formation of controlled, 3D cellular networks is desirable^[^ ^50^ ^]^ (Figure 3J–L).

While the exceptional capabilities of SLA in terms of accuracy and resolution are unquestioned, this printing strategy suffers from several weaknesses that deserve attention. In extrusion and inkjet‐based printing, for example, the materials of choice are selectively deposited at specific spatial locations. In contrast, SLA is traditionally based on the selective curing of a single, homogenous photoreactive material. This significantly limits the applicability of this method to the fabrication of structures with low‐compositional complexity, that is, a single bioink. As demonstrated, multicomponent structures can be fabricated by manually exchanging the photoreactive material between projections.^[^ ^43^ ^]^ However, such non‐continuous fabrication processes can be tedious, slow, and inaccurate. Another consideration that needs to be accounted for, especially in the context of 2PP, is the process throughput. The highly confined region of polymerization, which endows this fabrication method with its phenomenal accuracy, also imposes an extended process duration. This limits 2PP‐fabricated structures to the millimeter range, and even in this scale, fabrication can require days to accomplish.^[^ ^51^, ^52^, ^53^ ^]^ Recent works, however, indicate a trend toward the development of faster 2PP printing platforms, as discussed below.

### 2.3 Making It Fast

As mentioned above, the presence of living cells constitutes a limiting factor in bioprinting, profoundly narrowing the range of compatible materials and fabrication conditions. Moreover, as a rule, the conditions required to support long‐term cell viability cannot be optimally maintained during printing. For this reason, the cells need to be transferred as fast as possible to an environment that supports their metabolic demands, with a replenishing supply of oxygen and nutrients. Printing time, which is derived from the printing resolution, printout composition, object size, and fabrication technique, is thus a critical parameter that may directly impact the fate of the incorporated cells. The significance of printing duration is especially prominent in the generation of large, volumetric constructs composed of numerous thin layers. Projection‐based stereolithography presents a huge advantage over direct‐write SLA and extrusion‐based printing, as it enables fabrication in a layer‐at‐once fashion instead of tracing a set of coordinates for each layer.^[^ ^12^, ^40^, ^54^ ^]^ While using this strategy spares a considerable amount of processing time, it is still based on the conventional approach of consecutive material layering.

**Figure 4:**
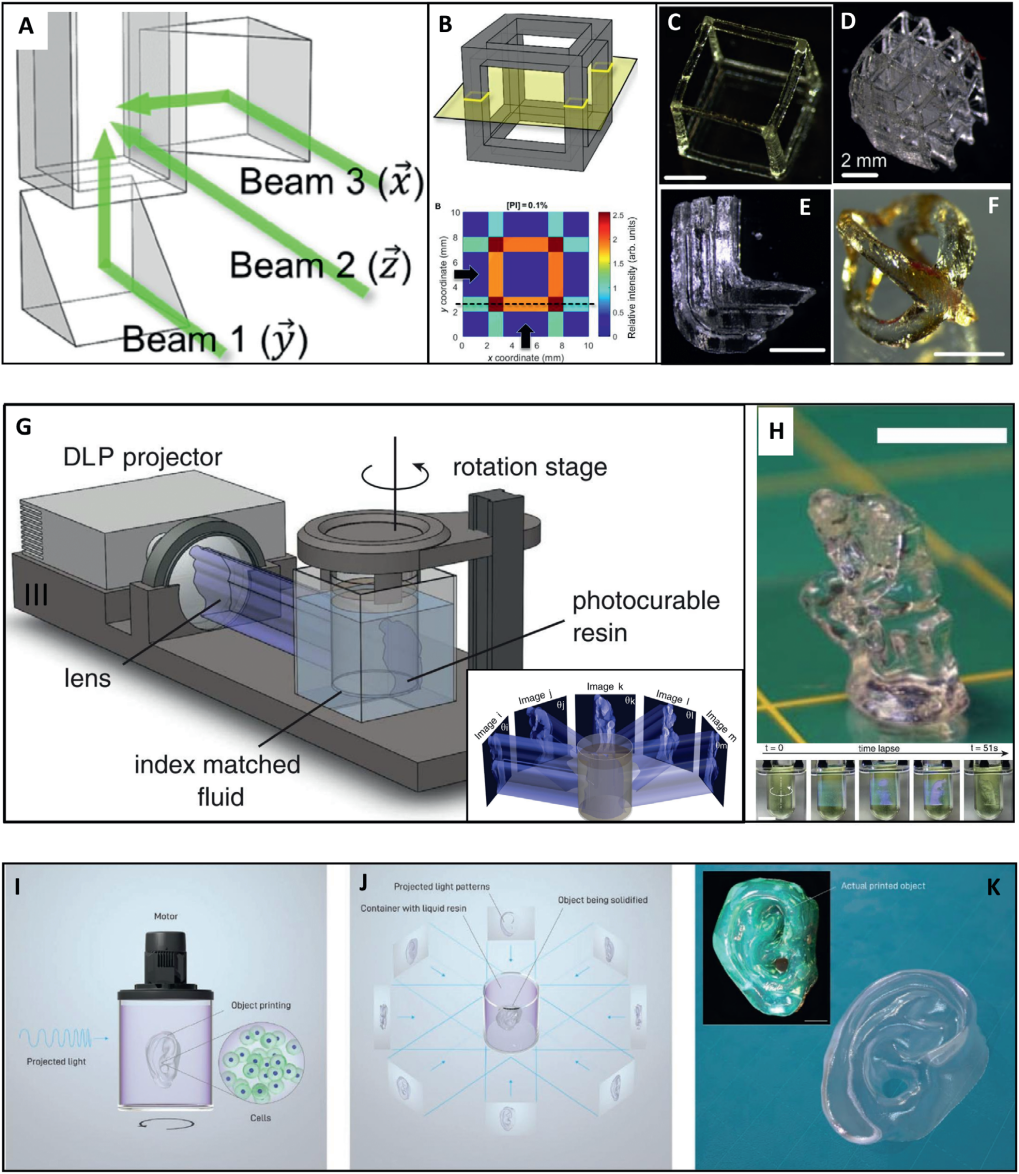
High‐speed volumetric printing. Holographic 3D fabrication. A) Prism mirrors direct beams at orthogonal directions into a photo‐sensitive resin that B) is consequently cured at the region of intersection. This results in generation of 3D shapes C–F) by a single short exposure of up to 10 s. Scale bars: 2 mm. Adapted with permission.^[^ ^55^ ^]^ Copyright 2017, AAAS. Computed axial lithography (CAL). G) Graphical illustration of the CAL approach. A set of 2D images is projected through a rotating tank filled with photo‐sensitive material. The superposition of exposures from multiple rotational angles eventually reaches an energy dose that is sufficient for curing the geometry of choice. H) The printed object, generated in less than 1 min, after extraction from the uncured material. A sequential view of the process is presented at the bottom. Scale bars: 10 mm. Adapted with permission.^[^ ^56^ ^]^ Copyright 2019, AAAS. Tomographic volumetric bioprinting. I) A cell‐laden biocompatible resin in a rotating tank is J) projected by 2D light patterns from multiple rotational angles. K) The resin then solidifies in selected areas where the accumulative absorbed dose overcomes a gelation threshold (Main: structure rendering. Inset: the actual printed structure). Scale bar: 2 mm. Reproduced with permission.^[^ ^58^ ^]^ Copyright 2019, Wiley‐VCH.

Recently, a new paradigm in photopolymer‐based additive fabrication has been proposed by Spadaccini and colleagues, enabling the fabrication of 3D geometries on a time scale of seconds.^[^ ^55^ ^]^ This incredible processing speed is achieved by the superposition of patterned optical fields from multiple beams, projected at orthogonal directions into a photo‐sensitive resin. The region in which the beams intersect defines the object's geometry, where the energy of the absorbed light overcomes a curing threshold. Using this unique holographic patterning system, a variety of 3D shapes made of PEGDA have been fabricated by a single light exposure of up to 10 s (**Figure** **4**A–F). These structures, however, were limited in their geometry due to the prismatic nature of the overlapping beams.^[^ ^55^ ^]^ To overcome these limitations, another novel approach denoted as “computed axial lithography” (CAL), has been developed. This technique, pioneered by Taylor and co‐workers, enables ultra‐fast printing of large and geometrically complex objects within a matter of seconds.^[^ ^56^ ^]^ This was achieved by using an innovative volumetric printing approach inspired by the image reconstruction procedures of computed tomography. The method is based on the concurrent printing of all points within a given 3D geometry by projecting a set of 2D images through a rotating tank containing a photo‐sensitive resin. The superposition of exposures from multiple rotational angles eventually reaches an energy dose that is sufficient for curing the geometry of choice. The non‐crosslinked photo‐sensitive resin is then washed away, leaving behind a solid 3D printout (Figure 4G,H). In addition to speed, this unique “volume‐at‐once” type of fabrication also offers several advantages over layer‐based printing. First, the fabricated objects present a smooth surface and are devoid of anisotrophic mechanical performance that may result from material layering. Second, the technique can be applied on high‐viscosity fluids and even on solids, such that the cured structure remains embedded in the surrounding material with minimal relative motion between the two. This eliminates the need to support the structure during the fabrication process, enabling the printing of bridges, overhanging elements, and disconnected parts. Third, the technique allows printing around preexisting objects, enabling the incorporation of external elements into the fabricated construct. Using this technique, the group demonstrated the fabrication of a large array of geometries and centimeter‐scale objects made of acrylate polymers and GelMA. The structures, containing features as small as 300 µm, were fabricated in an extremely short time frame of 30–300 s.^[^ ^56^ ^]^ A later publication by Loterie et al. demonstrated tomographic volumetric printing of acrylic and silicone parts with improved geometric fidelity. This was achieved by using an optimized projection source with an integrated feedback system, allowing high‐resolution fabrication of centimeter‐scale objects with features as thin as 80 µm in less than 30 s.^[^ ^57^ ^]^ In an intriguing study, Bernal et al. demonstrated the use of such volumetric printing techniques to biofabricate structures that are difficult to reproduce through conventional AM processes^[^ ^58^ ^]^ (Figure 4I–K). Using GelMA as a photocurable resin, the group printed a fluidic ball‐cage valve with free‐floating elements and a human auricle model, both of which were fabricated in less than 23 s. To prove the biocompatibility of the system, an anatomical trabecular bone model loaded with mesenchymal stromal cells was generated. This living construct, which contained an interconnected porous network, was reproduced with the smallest resolved feature measuring ≈145 µm. Printing speed was extremely high, with less than 13 s being required to complete the fabrication of an 8.5 × 9.3 mm structure. High cell‐viability of more than 85% was maintained throughout a 7‐day period, during which the culture was primed with osteogenic medium so as to mimic the osteoblast population within native bone. Vascular endothelial cells were then introduced into the pore network of the structure, leading to the formation of early angiogenic sprouts that began to invade the bone compartment of the construct. Finally, to assess the capacity of printed cells to synthesize new tissue matrix, the researchers fabricated a meniscus‐shaped implant in which articular chondroprogenitor cells were encapsulated at a density of 10^7^ cells mL^−1^. The cells, which exhibited high long‐term viability and metabolic activity, were found to synthesize neo‐ECM. This newly synthesized matrix increased the compressive modulus of the graft from ≈15 to ≈265 kPa, comparable to native human fibrocartilage.^[^ ^58^ ^]^

Altogether, these innovative volumetric printing schemes, which allow the fabrication of large, geometrically complex structures at unimaginably high speeds, are nothing less than game changers. Importantly, the ability to generate such constructs with densely packed, viable cells is an important milestone and a significant breakthrough in TE. Without a doubt, this technology is expected to play a central role in modern biofabrication, with far‐reaching implications on future developments and applications. It shares, however, a major drawback with the other above‐mentioned photopolymerization‐based printing techniques. Namely, as volumetric printing is based on the selective curing of a single type, homogenous, pre‐casted material, the printed construct inevitably presents low compositional complexity.

## 3 Future Perspectives

TE has taken enormous steps forward in recent years, with the latest advances in biofabrication techniques being a major driving force. The progress that has been made and the innovations described above address important aspects and bottlenecks in the field, speeding up its evolution. They also, however, reveal new complications to be overcome and further raise the bar for future developments. In the sections below we discuss potential directions for progress in the 3D bioprinting domain. An outlook on the impact of this emerging discipline on next‐generation research and medicine is also brought and discussed.

### 3.1 What Is in the Pipeline?

**Figure 5:**
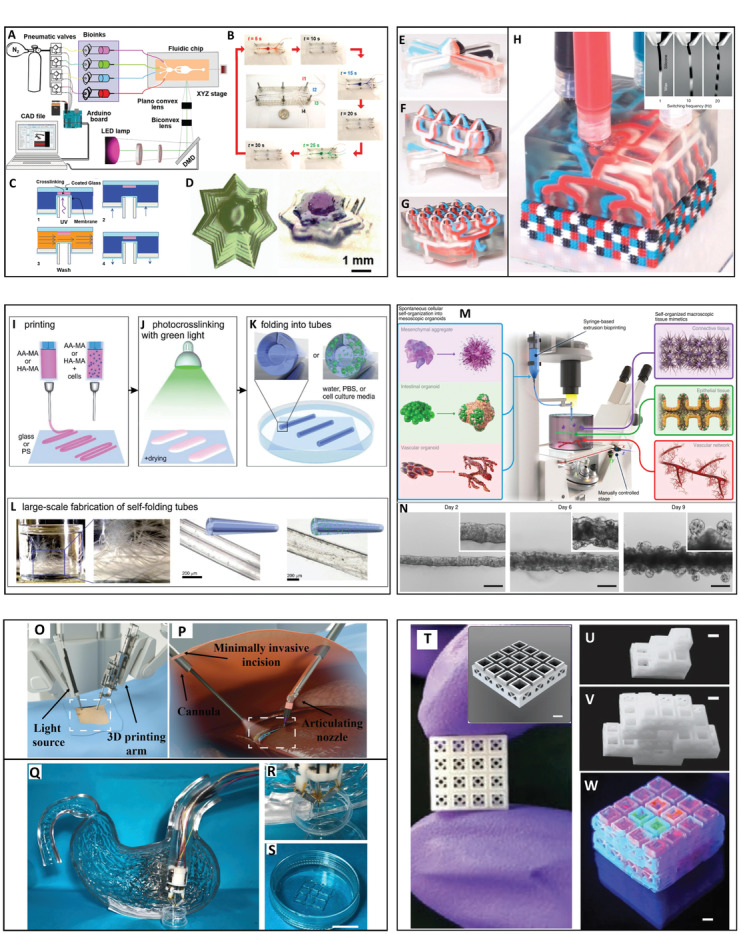
Emerging concepts. A stereolithographic 3D bioprinting platform with an integrated microfluidics device designed for fabrication of multimaterial and multicellular microstructures. A) Illustration of the setup. B) Operation of the microfluidics device that enables quick switching between different bioinks with intermediate washing steps. C) Schematics of the cyclic, 4‐steps bioprinting process inside the microfluidics chip. D) A single component and a three‐component structure made of PEGDA. Adapted with permission.^[^ ^59^ ^]^ 2018, Wiley‐VCH. Multimaterial, multinozzle 3D printing of voxelated matter. E) Four‐material printheads with a single nozzle, F) four nozzles at a 1 × 4 1D setup, and G) 16 nozzles at a 4 × 4 2D setup. H) Voxalated matter is extruded from a four‐material, 2D printhead with 4 × 4 nozzle setup. Inset: Operation of a two‐material nozzle that produces a continuous voxelated filament at different material switching frequencies. Adapted with permission.^[^ ^62^ ^]^ Copyright 2019, Springer Nature. 4D bioprinting of shape‐transforming structures. I) Layers of printed acellular or cell‐containing shape‐morphing hydrogels J) undergo photo‐crosslinking and mild drying and K,L) instantly fold into tubes upon immersion in aqueous media. Reproduced with permission.^[^ ^66^ ^]^ Copyright 2017, Wiley‐VCH. Bioprinting‐assisted tissue emergence (BATE). M) Illustration of the BATE concept. The fabrication process is based on deposition of high‐density cell suspensions into liquid precursors of ECM hydrogels that facilitate effective cellular self‐organization into macrostructures. N) Tube evolution of BATE‐printed intestinal tissue with lumen and budding structures formed at day 6 and crypts at day 9. Scale bars: 200 µm. Adapted with permission.^[^ ^70^ ^]^ Copyright 2020, Springer Nature. Endoscopic additive manufacturing. O,P) Illustration of the intracorporeal TE concept in which 3D printing is performed on the patient's internal organs by minimally invasive procedures using miniaturized printing platforms. Adapted with permission.^[^ ^74^ ^]^ Copyright 2020, IOP. Q–S) A microbioprinting platform can be installed on an endoscope to treat gastric wall injuries. Scale bar: 1 cm. Adapted with permission.^[^ ^75^ ^]^ Copyright 2020, IOP. T–W) Printed stackable microcage modules for manual assembly. Printed rigid stackable microcage scaffolds with 1 × 1, 2 × 2, and 4 × 4 designs can be manually assembled and scaled to adopt a desired geometry. Additionally, each microcage can be loaded with a cargo of choice, such as cells and/or therapeutics (demonstrated in (W) using fluorescent microgels). Scale bars: 1.5 mm. Adapted with permission.^[^ ^79^ ^]^ Copyright 2020, Wiley‐VCH.

Obviously, current biofabrication protocols are far from providing the capacity to generate transplantable, functional, complex tissues and organs. From a technical point of view, this may result, in part, from the fact that each fabrication method is characterized by an inherent set of strengths and weaknesses. That is to say, a technique that excels in fabricating specific types of materials and structures will probably give sub‐optimal results for different kinds of compositions and geometries. As discussed, tissues and organs are generally composed of an assortment of cells, materials, and architectures. Thus, low efficiency and/or reduced performance and building quality are to be expected during the fabrication of some elements of the final printout. With this in mind, it is reasonable to expect future 3D bioprinting developments in which attempts will be made to broaden the applicability of existing fabrication protocols. Indeed, scientists have already begun to develop modified printing schemes that compensate, to some extent, for the inherent shortcomings that characterize their underlying working principles. For example, stereolithographic bioprinting can give excellent results in terms of accuracy. However, as mentioned, it usually yields constructs that are made of a single bioink. To address this limitation, the printing device may be re‐configured to enable easy and rapid in‐process exchange of the photocurable resin. Such a configuration has been proposed by Khademhosseini and colleagues, who developed a stereolithographic bioprinting platform with an integrated microfluidics device. The novel system enables projection‐based printing with the option to quickly and efficiently switch between different bioinks during the process. Using this automated system, multimaterial and multicellular microstructures and biomimetic heterogeneous tissue constructs were continuously fabricated, at high‐resolution, within a time‐frame of seconds^[^ ^59^ ^]^ (**Figure** **5**A–D). In a later work, Mayer et al. demonstrated the use of a microfluidics system integrated into a 2PP‐based laser lithography apparatus. Using this setup, the authors printed multimaterial, fluorescent, 3D security features based on four emission colors. While this research did not assess the functionality of the system for working with biomaterials and cells, it elegantly proved that integration with microfluidic systems can also greatly increase the complexity of 2PP‐printed structures.^[^ ^60^ ^]^

As with compositional complexity, improvements in printing speed can also dramatically broaden the applicability of fabrication methods that do not excel in terms of throughput. For instance, the production rate of the accurate (yet slow) 2PP method can be greatly enhanced if polymerization is executed in a layer‐by‐layer, instead of point‐by‐point, fashion. This concept was realized in a work conducted by Saha et al.^[^ ^61^ ^]^ In this study, the performance of a novel parallel process, based on femtosecond projection, was compared to the commonly implemented point‐by‐point writing scheme. Using layer‐by‐layer projection of digital masks, the group succeeded in increasing the throughput up to three orders of magnitude compared to that achieved by existing serial techniques. Importantly, the improved printing rate, reaching 8.7 mm^3^ h^−1^, was attained without compromising the characteristic 2PP sub‐micrometer resolution.^[^ ^61^ ^]^

In addition to 2PP printing techniques, extrusion‐based fabrication procedures would benefit from improved process throughput, especially when applied to the construction of large objects. This can be achieved, for example, by parallelizing several multimaterial deposition processes. An intriguing approach in this direction was presented in a recent study by Lewis and colleagues.^[^ ^62^ ^]^ The group developed a unique setup in which a single printhead is capable of depositing up to eight different materials (modeled in this work by silicone, wax, epoxy, and gelatin‐based inks). The different materials flow through independent channels that eventually merge into a single ink flow, immediately before the nozzle outlet. High‐frequency switching between the printing materials allows extrusion of filaments composed of distinct volume elements (voxels) along their length. When adjacently deposited, in a layer‐by‐layer manner, a multimaterial 3D structure is formed, with a voxel volume approaching that of the nozzle diameter cubed. The printing heads can also be designed to contain multiple nozzles as a 1D array (e.g., 4 nozzles in a 1 × 4 setup) or 2D array (e.g., 16 nozzles in a 4 × 4 setup) (Figure 5E–H). This multimaterial, multinozzle design thus considerably boosts printing throughput, not only by avoiding the need for an individual printhead for each material, but also by parallelizing the fabrication process. To demonstrate the performance of this setup, a soft robotic walker equipped with sixteen 12 mm x 12 mm x 17 mm actuators was printed within 17 min using stiff and flexible silicone inks.^[^ ^62^ ^]^

Another strategy for speeding up extrusion‐based fabrication processes may be based on our vision of an “inside‐out” printing scheme. In this hypothetical mechanism, the object is simultaneously fabricated by multiple three‐axis controllable dispensing tips that follow distinct, non‐intersecting paths. In contrast to the canonical printing scheme, the fabrication begins from the core of the object and continues, in a layer‐by‐layer fashion, toward its periphery. This process is theoretically feasible due to the presence of a support medium that envelops the extruded material and holds it in place, simulating printing in a zero‐gravity environment. By printing inside a support bath that is considerably larger than the printout, each dispensing needle can approach the object from a different angle, including from the bottom. In this way, the fabrication time of large, volumetric structures could be considerably reduced as a function of the number of simultaneously operated dispensing tips.

While boosting the processing speed is highly advantageous, the major limitation of extrusion‐based 3D fabrication is the printing resolution. As discussed above, the intuitive strategy of decreasing the diameter of the dispensing tip is limited due to the increasing shear stress, to which the cells will eventually succumb. Thus, in this case, alternative, out‐of‐the‐box thinking is highly desired. An interesting approach would be to use “smart materials” as inks for the fabrication of structures that can transform their shape in response to stimuli. Such a technique, denoted “4D printing,” could be utilized for the fabrication of structures with an attainable resolution using a standard extrusion‐based printer. Upon stimulation, however, the printout would undergo a structural transformation to attain dimensions that are beyond the building capability of the underlying fabrication method.^[^ ^6^, ^63^, ^64^, ^65^ ^]^ A proof for the feasibility of this approach was provided by Kirillova et al., who used photo‐crosslinkable methacrylated alginate and hyaluronic acid as shape‐morphing hydrogels.^[^ ^66^ ^]^ The materials were loaded with cells and used as bioinks for the extrusion‐based printing of 2D, rectangular shapes. Following photo‐crosslinking at 530 nm, mild drying, and immersion in aqueous media, the printed layers instantly folded into tubes with an internal diameter of as low as 20 µm (Figure 5I–L). This value is on the scale of the internal diameters of the smallest blood vessels, the geometries of which are extremely challenging to reproduce using existing extrusion‐based printing techniques. Notably, neither the printing process nor the post‐printing treatment adversely affected the cells that survived for at least 7 days without any decrease in their viability.^[^ ^66^ ^]^

Another strategy for overcoming the limitations of using a particular fabrication technique is to synergistically combine several complimentary printing schemes into a single platform, whereby the strengths of one cover for the weaknesses of the other. An intriguing example of the implementation of such a strategy has been presented by Shanjani et al.^[^ ^67^ ^]^ In this work, PSL and extrusion‐based printing techniques were combined for the fabrication of complex, multimaterial cellular constructs. The structures were composed of extruded, thermoplastic PCL that formed a porous, rigid scaffold, combined with soft, photo‐crosslinkable PEGDA hydrogel that contained living endothelial cells and mesenchymal stem cells. The fabrication was based on a repeating process in which strands of molten PCL were deposited on the build platform, followed by immersion into the pre‐polymer solution and photo‐curing of the regions that needed to be gelled. Using this scheme, various complex designs were generated, including cellular scaffolds with integrated perfusable conduits.^[^ ^67^ ^]^ For more information and insights on such multi‐technological, hybrid fabrication methods, we recommend the readers to peruse these two recently published articles.^[^ ^68^, ^69^ ^]^

Aside from improving established printing methods, or combining them into integrated platforms, the future of the field also depends on the development of new 3D biofabrication techniques. While not in the scope of this review, it is worth mentioning that the last several years have been characterized by the emergence of a variety of innovative printing schemes and concepts. These include, among others, procedures that involve magnetic and acoustic‐based printing, electrohydrodynamic processing, and new methods for the 3D patterning of spheroids/organoids. Most of these techniques are still in their infancy and require further development and tuning. Nevertheless, a taste of their performance can already be obtained from recently published works.^[^ ^9^, ^68^, ^69^ ^]^ An intriguing example of such a technique was recently presented by Lotolf and colleagues.^[^ ^70^ ^]^ In their work, organoid‐forming stem cells were used as building blocks that can spatially self‐arrange according to a pre‐defined geometry. The process was based on the deposition of high‐density cell suspensions into liquid precursors of ECM hydrogels that facilitated effective cellular self‐organization. Using this approach, termed bioprinting‐assisted tissue emergence, centimeter‐scale epithelial, connective, and vascular tissues were fabricated. Importantly, the printed biostructures were characterized by native‐like features such as lumens, crypts, and branches and responded to chemical stimuli, indicating their high physiological relevance^[^ ^70^ ^]^ (Figure 5M,N).

Also worth mentioning is the evolving approach of patient‐specific in situ 3D printing, in which constructs are printed, in vivo, directly at the target site.^[^ ^71^, ^72^, ^73^ ^]^ A subset of this approach, the newly emerged concept of intracorporeal 3D printing, or endoscopic AM, is performed by minimally invasive procedures using miniaturized printing platforms^[^ ^74^, ^75^ ^]^ (Figure 5O–S). Either way, as the constructs are fabricated on or inside the patient's body, which serves as a living bioreactor, there is no need for an in vitro maturation phase. Another approach that targets a clinical need is the production of “off‐the‐shelf” tissue substitutes. At the heart of this concept is the ambition to provide clinicians with a pool of available, readily transplantable, pre‐prepared, engineered body parts. The advantage of this approach is clear: malfunctioning tissue might be repaired or replaced without going through tedious preliminary design and manufacturing processes. One of the major obstacles in this concept, however, is the limited capacity to personalize the pre‐prepared tissue so that it matches the patient, both structurally and immunologically. Currently, resolving the problem of immune rejection of cell‐containing implants requires complicated procedures (i.e., cellularization of the implant with patient‐derived cells,^[^ ^76^ ^]^ Human Leukocyte Antigens (HLA) matched^[^ ^77^ ^]^ or engineered, “universal,” hypoimmunogenic cells^[^ ^78^ ^]^). The process of structural matching, on the other hand, could be significantly simplified. This could be done, for example, by enabling the clinician to produce patient‐specific geometries from pre‐printed building blocks without the need for special equipment or long training. Such an approach was elegantly demonstrated by Subbiah et al.^[^ ^79^ ^]^ The group used lithography‐based 3D printing to construct a microcage scaffold assembly system for regeneration of hard tissues. The rigid, miniaturized, stackable microcage modules could be manually assembled and scaled by the user to generate the required geometry. Moreover, as each module is amenable to loading with a cargo of choice, cells and therapeutic agents could be patterned in 3D within the composed construct^[^ ^79^ ^]^ (Figure 5T–W).

Finally, it should be pointed out that the described progress and future advances should go hand in hand with the continuous improvement of printing materials, design tools, process algorithms, and post‐printing culturing and maturation techniques. While not thoroughly discussed in this review, it must be remembered that these elements are inseparable from the printing process. Information on the latest advances in these important disciplines can be found in recent reviews.^[^ ^8^, ^80^, ^81^, ^82^, ^83^, ^84^ ^]^

A summary table that presents some of the key features of the printing methods covered in this review can be found below (**Table** **1**).

**Table 1:** Key features of the printing methods covered in this review

Ref.	Technique	Materials	Cells	Printed structures	Strengths	Weaknesses
Liu et al.^[^ ^21^ ^]^	Continuous multimaterial extrusion	Nanosilicate, hydroxyapatite, carbon nanotubes, DNA, PEGDA/GelMA/alginate‐based bioinks, Pluronic F‐127 (as a supporting medium for embedded printing)	Mouse preosteoblasts (MC3T3‐E1), human dermal fibroblasts (HDFs), human hepatocytes (HepG2), human mesenchymal stem cells (hMSCs), human umbilical vein endothelial cells (HUVECs)	Multicomponent and multi‐cell structures, bioelectronics, gradient structures	High compositional complexity, smooth switching between bioinks, simultaneous extrusion of different bioinks, faster than multi‐printhead extrusion printers	Low shape fidelity when printing large multi‐layered structures; limited resolution in the presented setup; the multi‐capillary configuration prevents printing of different inks under different temperatures
Skylar–Scott et al.^[^ ^29^ ^]^	Extrusion, sacrificial writing into functional tissue (SWIFT)	Rat tail collagen I, Matrigel, gelatin (as a sacrificial ink)	Personal genome project 1 (PGP1) or BJFF iPSCs and derived differentiated cells, HUVECs	Perfusable constructs and anatomical structures with high cellular density	High structural complexity, free‐form printing of vascular networks, generation of organ‐specific tissues with native cell density	The construct's design is limited in terms of the geometry and composition of its non‐sacrificial component; a second step of post‐printing perfusion needs to be introduced into the fabrication scheme in order to obtain cell‐lined channels
Homan et al.^[^ ^30^ ^]^, Lin et al.^[^ ^31^ ^]^	Extrusion, sacrificial writing into ECM	Gelatin, fibrin, poly(ethylene oxide), Pluronic F‐127 (as a sacrificial ink)	Human immortalized proximal tubule epithelial cells (PTECs, RPTEC/TERT1), human primary RPTEC, human renal carcimoma cells (A498), human neonatal dermal fibroblasts (HNDF), glomerular microvascular endothelial cells (GMECs)	Perfusable anatomical structures	High structural complexity, free‐form printing of vascular networks	The construct's design is limited in terms of the geometry and composition of its non‐sacrificial component; a second step of post‐printing perfusion needs to be introduced into the fabrication scheme in order to obtain cell‐lined channels
Kang et al.^[^ ^32^ ^]^	Extrusion, multidispensing modules for delivering cell‐laden hydrogels together with synthetic biodegradable polymers, "integrated tissue–organ printer (ITOP)"	Gelatin, fibrinogen, hyaluronic acid, glycerol, poly(ɛ‐caprolactone) (PCL, as a support), pluronic F‐127 hydrogel (as a sacrificial material)	3T3 fibroblasts, C2C12 myoblasts, human amniotic fluid‐derived stem cells (hAFSCs), rabbit primary auricular chondrocytes	Cellular tissue constructs and anatomical structures	High structural complexity; generation of structurally stable multi‐layered constructs	The supporting PCL, which remains an integral part of the construct, cannot be loaded with or penetrated by living cells. It may also introduce non‐physiological, extra‐rigidity into the constructs; prolonged fabrication time (for large constructs)
Bhattacharjee et al.^[^ ^33^ ^]^	Extrusion into a support particulate gel	Carbopol ETD 2020 polymer (as a support material), polyvinyl alcohol (PVA), polydimethylsiloxane (PDMS), polyacrylamide, polyethylene glycol, hyaluronic acid, sodium alginate, acid‐solubilized bovine collagen	Human aortic endothelial cells (HAECs), Madin–Darby canine kidney (MDCK) cells, MCF10A epithelial cells	Structurally complex acellular 3D objects, thin closed shells and hierarchically branched tubular networks, cellular tubular structures	Very high structural complexity; free‐form printing; high transparency and thermostability of the support medium	Extraction of the printout cannot be executed by cell‐friendly, delicate procedures that involve liquefaction or degradation of the support; prolonged fabrication time (for large constructs)
Hinton et al.^[^ ^34^ ^]^, Lee et al.^[^ ^35^ ^]^	Extrusion into a support particulate gel, "freeform reversible embedding of suspended hydrogels" (FRESH)	Gelatin (as a support material), alginate, hyaluronic acid, fibrinogen, acid‐solubilized rat tail collagen, acidified bovine collagen, Matrigel	MC3T3‐E1.4 fibroblasts, C2C12 myoblasts, HES3‐human embryonic stem cell‐derived cardiomyocytes, human ventricular cardiac fibroblasts, HUVECs	Structurally complex acellular and cell‐containing anatomical structures, perfusable vascular constructs	Very high structural complexity; free‐form printing; extraction of the printouts is performed by a simple, delicate, and rapid procedure (heating to 37 °C)	The support material is heat‐sensitive (which restricts the use of printing materials that cure under elevated temperature); the support medium is semi‐transparent; prolonged fabrication time (for large constructs)
Kupfer et al.^[^ ^36^ ^]^	Extrusion into a support particulate gel, "freeform reversible embedding of suspended hydrogels" (FRESH)	Gelatin (as a support material), gelatin methacrylate (GelMA), collagen methacrylate	Human induced pluripotent stem cells (hiPSC)	Structurally complex, cell‐containing perfusable anatomical structures	Very high structural complexity; free‐form printing; extraction of the printouts is performed by a simple, delicate, and rapid procedure (heating to 37 °C)	The support material is heat‐sensitive (which restricts the use of printing materials that cure under elevated temperature); the support medium is semi‐transparent; prolonged fabrication time (for large constructs)
Shapira et al.^[^ ^37^ ^]^, Noor et al.^[^ ^38^ ^]^	Extrusion into a hybrid support medium	Alginate, xanthan gum (as support materials), neutral pepsin treated bovine collagen, neutral pepsin treated decellularized pig and human omentum	NIH/3T3 fibroblasts, HUVECs, neonatal rat cardiac cells, hiPSC‐derived cardiomyocytes, and endothelial cells	Structurally complex, cell‐containing perfusable anatomical structures	Very high structural complexity; free‐form printing; high transparency and thermostability of the support medium, extraction of the printouts is performed by delicate, cell‐friendly procedures	Extraction of the printout requires the addition of external reagents. It may also take longer to accomplish in comparison to extraction from thermoreversible supports
Ma et al.^[^ ^43^ ^]^	Two‐step, projection‐based stereolithography	GelMA, glycidal methacrylate‐hyaluronic acid (GMHA)	hiPSC‐derived hepatic progenitor cells (hiPSC‐HPCs); HUVECs, adipose‐derived stem cells (ADSCs)	Cellular anatomical microstructures	High resolution and accuracy; fast, layer‐at‐once fabrication; sequential, multi‐step procedure enables fabrication of constructs with high compositional complexity	Suitable for fabrication of relatively thin constructs; the non‐continuous, manual exchange of the photoreactive material between projections limits the capacity to fabricate complex, thick multi‐layered structures
Yu et al.^[^ ^44^ ^]^	Projection‐based stereolithography	GelMA, pepsin treated decellularized pig's heart and liver	hiPSC‐derived cardiomyocites and hepatocytes	Cellular anatomical microstructures	High resolution and accuracy; fast, layer‐at‐once fabrication	Suitable for fabrication of relatively thin constructs; the printed structures present low compositional complexity (the fabrication process is based on selective curing of a single type, homogenous photoreactive material)
Grigoryan et al.^[^ ^45^ ^]^	Projection‐based stereolithography, "stereolithography apparatus for tissue engineering" (SLATE)	Poly(ethylene glycol) diacrylate (PEGDA), GelMA	Human mesenchymal stem cells (hMSCs), human lung epithelial cells (A549), human lung fibroblasts (IMR‐90), HUVECs, normal human dermal fibroblasts (NHDFs), rat primary hepatocytes, human red blood cells (RBCs)	Intricate vascular architectures with functional internal topologies, bioinspired models of natural topologies, structurally complex perfusable engineered tissues	Accurate and rapid fabrication of acellular or cellular hydrogels containing intricate and functional vascular architectures	The printed structures present low compositional complexity (the fabrication process is based on selective curing of a single type, homogenous photoreactive material)
Worthington et al.^[^ ^48^ ^]^, Thompson et al.^[^ ^49^ ^]^	Two‐photon polymerization	IP‐S photoresist; photopolymerizable poly(caprolactone) (PCL)	hiPSC‐derived retinal progenitor cells (RPCs)	Cell‐containing porous scaffold	Very high printing resolution and accuracy	Low process throughput limits the fabricated structures to the millimeter and sub‐millimeter range; the printed structures present low compositional complexity (the fabrication process is based on selective curing of a single type, homogenous photoreactive material); cells are introduced into the structure in a separate, post‐printing step
Larramendy et al.^[^ ^50^ ^]^	Two‐photon polymerization	IP‐L 780 photoresist, collagen (as a coater for the printed scaffold)	Neuron‐like PC12 cells	Stackable cell‐microcages	Very high printing resolution and accuracy (crucial for fabrication of cell‐size microcontainers)	Low process throughput limits the fabricated structures to the millimeter and sub‐millimeter range; the printed structures present low compositional complexity (the fabrication process is based on selective curing of a single type, homogenous photoreactive material); cells are introduced into the structure in a separate, post‐printing step
Shusteff et al.^[^ ^55^ ^]^	One‐step, multi‐beam volumetric printing, holographic patterning	PEGDA		Acellular 3D geometries	Extremely fast (almost instantaneous) fabrication by a single light exposure; the whole structure is fabricated "at once"—no reliance on material layering or support	The fabricated structures are limited in their geometry due to the prismatic nature of the overlapping beams; the printed structures present low compositional complexity (the fabrication process is based on selective curing of a single type, homogenous photoreactive material); lower resolution and accuracy in comparison to the more "conventional", stereolithographic methods
Kelly et al.^[^ ^56^ ^]^, Loterie et al.^[^ ^57^ ^]^, Bernal et al.^[^ ^58^ ^]^	"Computed axial lithography" (CAL), tomographic volumetric printing	Bisphenol A glycerolate (1 glycerol/phenol) diacrylate (BPAGDA), PEGDA, GelMA, di‐pentaerythritol pentaacrylate (SR399), photocurable thiol‐ene silicone resin	Equine‐derived articular chondroprogenitor cells (ACPCs), human bone marrow‐derived mesenchymal stromal cells (MSCs), human endothelial colony forming cells (ECFCs)	Large and geometrically complex cellular and acellular 3D objects and anatomical structures	Extremely fast fabrication; the whole structure is fabricated "at once"—no reliance on material layering or support; allows printing around pre‐existing objects	The printed structures present low compositional complexity (the fabrication process is based on selective curing of a single type, homogenous photoreactive material); lower resolution and accuracy in comparison to the more "conventional", stereolithographic methods
Miri et al.^[^ ^59^ ^]^	Microfluidic‐integrated, multimaterial projection‐based stereolithography	PEGDA, GelMA	HUVECs, human mesenchymal stem cells (MSCs), human dermal fibroblasts, NIH/3T3 fibroblasts, MCF7 breast cancer cells, C2C12 skeletal muscle cells	Acellular 3D geometries, cellular tissue constructs, and biostructures	High resolution and accuracy; fast, layer‐at‐once fabrication; automated procedure enables fabrication of multi‐layered constructs with high compositional complexity	Current design is limited to fabrication of small‐sized objects
Mayer et al.^[^ ^60^ ^]^	Microfluidic‐integrated, multimaterial two‐photon polymerization	Quantum dots and ATTO dyes containing photoresists		Acellular multimaterial 3D microstructures	Very high printing resolution and accuracy; high compositional complexity; all the steps and components that are required for fabrication are integrated into one machine	Low process throughput limits the fabricated structures to the millimeter and sub‐millimeter range
Saha et al.^[^ ^61^ ^]^	Projection‐based, layer‐by‐layer parallelized two‐photon polymerization	Pentaerythritol triacrylate (PETA) and bisphenol A ethoxylate diacrylate (BPADA) based resists		Acellular 3D microstructures with nanoscale features	Very high printing resolution and accuracy; layer‐at‐once fabrication results in a much higher throughput than conventional point‐by‐point 2PP writing schemes	Low compositional complexity (the fabrication process is based on selective curing of a single type, homogenous photoreactive material)
Skylar‐Scott et al.^[^ ^62^ ^]^	Multimaterial multinozzle 3D (MM3D) extrusion‐based printing of voxelated matter	Silicone, wax, epoxy, and gelatin‐based inks		Acellular multimaterial large 3D structures and functional objects	Very high throughput due to parallel operation of multiple printheads, each is capable of extruding up to eight different materials; ability to print high‐viscosity inks	The presented setup is only capable of producing objects in periodic layouts; lower resolution in comparison to inkjet‐based 3D bioprinting (that generates voxelated 3D objects using low‐viscosity inks)
Kirillova et al.^[^ ^66^ ^]^	4D biofabrication	Methacrylated alginate and hyaluronic acid	Mouse bone marrow stromal cells (D1)	Hollow, self‐folding cellular and acellular tubes	Generation of structures with features that are extremely challenging to reproduce using existing extrusion‐based printing techniques	The described technique can be utilized to generate a relatively narrow range of geometries (limited freedom of design)
Shanjani et al.^[^ ^67^ ^]^	Extrusion‐based printing combined with projection‐based stereolithography, "Hybprinter"	PCL, PEGDA	HUVECs, C3H10T1/2 mouse mesenchymal stem cells	Cellular and acellular constructs and perfusable structures	Generation of structurally stable multi‐layered and multicomponent constructs	Relatively low resolution in comparison to other light‐based printing methods; the supporting PCL, which remains an integral part of the construct, cannot be loaded with or penetrated by living cells; it may also introduce non‐physiological, extra‐rigidity into the constructs
Brassard et al.^[^ ^70^ ^]^	Extrusion, organoid bioprinting into ECM, "bioprinting‐assisted tissue emergence" (BATE)	Neutralized bovine dermis collagen type I, Matrigel	C2C12, HUVECs, hMSCs, human intestinal stem cells (hISCs), mouse intestinal stem cells (mISCs), mouse intestinal mesenchymal cells (IMCs)	Cellular tissue‐like structures	Fast fabrication of centimeter‐scale tissues with native‐like features	Fabrication of structurally complex, multi‐layered thick constructs has not been demonstrated; gelling kinetics of the ECM hydrogel prevents long printing processes
Adib et al.^[^ ^74^ ^]^, Zhao et al.^[^ ^75^ ^]^	Intracorporeal/in situ in vivo extrusion‐based bioprinting	GelMA/laponite/methylcellulose (GLM); gelatin–alginate hydrogels	NIH/3T3 fibroblasts, human gastric epithelial cells (GES‐1), human gastric smooth muscle cells (HGSMCs)	Cellular and acellular lattice scaffolds	Printing at physiologically relevant conditions on soft, living tissue (inside the body); the printing can be performed by a micro bioprinting platform during a minimally invasive procedure	Currently limited to fabrication of low resolution constructs with low structural and compositional complexity
Subbiah et al.^[^ ^79^ ^]^	Projection‐based stereolithography	LithaBone TCP 300, GelMA	HUVECs, hMSCs	Stackable microgel‐loaded microcage modules	Generation of modular scaffolds that can be manually assembled and scaled by the user to match a required geometry; high compositional complexity could be achieved by loading with cargo of choice	The assembled scaffolds are of low resolution and structural complexity; the rigidity of the scaffolds limits their application to the regeneration of hard tissues

### 3.2 The Future of Printed Tissues and Organs—At the Crossroad of Reality

So, what should we expect to see in the near and far future? What will be the impact of the evolving 3D bioprinting field on modern healthcare, biotechnology, and academic research? In this section, we try to depict three hypothetical scenarios. Reality will most probably navigate its way somewhere in between.

The first is an ideal scenario for tissue engineers and is governed by technology and know‐how. That is to say, progress in the 3D biofabrication field will be dictated mainly by our capacity to build more advanced printing machines, formulate improved bioinks, and efficiently expand cells and culture the printed structures. In this scenario, the basic assumption is that biology will not pose an obstacle that cannot eventually be overcome on the journey toward engineered functional tissues and organs. Contrariwise, given a precise spatial positioning of the proper cells in meticulously formulated materials and under specific controlled conditions, the printed living components will organize and mature to form the desired structures. This does not mean that the cellular component of the engineered tissues will not require special preparation, guidance, and care. Rather, the biological knowledge that will be gained as the field evolves will suffice to fuel the progress. Under these hypothetical conditions, it is not too ambitious to assume that our ability to 3D fabricate basic, physiologically functional biostructures will mature in the foreseen future. Such a capacity will enable the production of the core constituents of animal and human tissues to a level at which most, or almost all, of the functionality of the native components is mimicked by the printed counterparts. Obviously, the progress must be accompanied by the development of advanced bioreactors and supporting accessories that enable controlled, long‐term cultivation of the living constructs. Such achievements will boost biological research, facilitating a much deeper investigation of the molecular, developmental, and physiological processes that are at the heart of life. They are also expected to revolutionize the fields of pharmacology and drug screening that currently rely on less reliable models such as 2D cell cultures, organ‐on‐a‐chip models, 3D non‐vascularized cellular constructs, and animals. Successful fabrication of 3D hierarchical tissue structures containing heterogeneous cell populations and supportive vasculature will gradually trigger attempts to use them for regenerative purposes. Animal models will first be used to prove the capacity of engineered tissues seeded with autologous cells to integrate into the host and to maintain long‐term activity. Follow‐up experiments will then be conducted to test whether a printed implant can regain the functionality of a defective tissue, or at least compensate, to some extent, for the loss of its activity. An array of integrated microsensors and actuators may be used to provide these crucial data, together with an indication of the tissue's activity and physiological state during maturation and post‐implantation. Such an integrated electronic system will work in a bi‐directional way, also allowing on‐demand intervention by electrical excitation or release of active compounds into the implant's surroundings.^[^ ^85^ ^]^ After confirming a therapeutic benefit in animal models, a race toward the development of clinical applications will start. First, cooperation will be established between, on one side, academia and the biotechnological industry and, on the other side, healthcare providers and hospitals. The latter will then set up their own bioprinting centers in which the whole process will take place. A typical procedure might begin with the harvest of cells and/or biomaterials from the patient, followed by their being processed into bioinks. Alternative sources of immune‐compatible cells, such as iPSC banks or “universal” iPSC lines, can also be used.^[^ ^76^, ^77^, ^78^ ^]^ In the next step, the engineered tissue will be fabricated on the basis of data extracted from 3D imaging of the patient's own anatomy, or from a generic model that will undergo personalized adaptations. When the integral sensory system and additional complementary assays indicate the maturation of the tissue, transplantation will be performed. The patient will then be continuously monitored by the healthcare provider with the assistance of wireless communication between the integrated electronics and an extracorporeal receiver. From this stage, the next significant step will be toward a higher level in the hierarchy, which is the level of the organ. Since constructs' volumes will greatly increase in the 3D bioprinting of full‐size human organs, the integration of ultra‐fast fabrication techniques may be required. Nevertheless, as speed will probably still come at the expense of printing resolution and complexity, such methods should be used in combination with other complementary, more accurate fabrication procedures. A representative scheme may be based on a hybrid platform in which an organ's parenchyma is fabricated at high speed around accurately pre‐printed organ‐specific microstructures and branched vascular system. After printing, the engineered organs will be connected to computer‐guided bioreactors that will constantly monitor their culturing environment and physiological status. The recorded data will be processed to generate a feedback loop that ensures a proper supply of oxygen, nutrients, essential biofactors, and external stimuli to the living organ. When the organ is functional and fully mature, it will be transplanted instead of, or in parallel to, its faulty natural counterpart, to regenerate function. Optionally, as discussed above, the engineered organs may be designed to maintain reciprocal communication with a medical specialist by virtue of integrated arrays of sensors and actuators. The integrated electronics may also be controlled by an internal feedback loop that can automatically intervene in the transplant's activity in cases of rapidly emerging, life‐threatening complications.

While the scenario depicts an optimal outcome, it presumably will not be realized in the near future. This is due to the long list of associated biological and technological challenges that will probably require prolonged research and development. An example of such a challenge is the current absence of efficient cell expansion techniques. The human adult heart, for instance, contains ≈4 billion muscle cells (CM). Hence, a huge number of these cells first needs to be attained in order to print a full size, transplantable, cellular organ. As adult human CM exhibit a very limited self‐renewal capacity, an enormous population of patient‐specific stem cells must first be established and differentiate accordingly. This requires execution of complicated procedures for attaining a highly pure CM culture with the proper phenotype. Unfortunately, these procedures, in their current form, are particularly costly and very demanding for scaling up.^[^ ^86^, ^87^, ^88^, ^89^, ^90^ ^]^ Another challenge that has largely stayed out of focus, is the innervation of engineered tissues and organs. While not essential for tissue organization and survival, its role in organ development, functionality, and regeneration is increasingly being recognized. Addressing this issue adds another layer of complexity that may require expanding both knowledge and laboratory practice.^[^ ^91^ ^]^ A wide perspective on the challenges presented by whole organ bioprinting and future directions for the field can be found in a recent comprehensive review.^[^ ^92^ ^]^

In the next hypothetical scenario, biology is much less cooperative. Referred to here as the “glass ceiling” scenario, it depicts a situation in which most of the complex engineered cellular constructs will not reach an adequate level of functional resemblance to the native tissue. In other words, although fabricated to precisely mimic the composition, architecture, and hierarchy of the native tissue, and albeit treated with the most updated differentiation and culturing protocols, the vast majority of printed tissues will display only limited functionality. Thus, while still being able to provide substantial benefits for research and biotechnological applications like basic drug screening, cultured meat, bioproduct production, etc., the non‐ideal performance of printed biostructures will prevent their clinical use. That being the case, what could be the reason that the engineered tissue does not organize and perform like a native one? If we precisely recapitulate the composition and spatial position of the tissue's elements, introduce the cells into a supportive environment and provide them with appropriate cues, what else is required for the formation of a native‐like, functional tissue? Two possible options are time and the sequence of events. The reason we choose to focus on these specific parameters is that they are prominent during natural development, but are not reflected, or taken into consideration, in current 3D bioprinting protocols. During the natural development of higher organisms, complex biological structures are progressively generated in time frames that are significantly longer than the course of an average 3D bioprinting session. These processes are also characterized by an orchestrated sequence of events with a defined hierarchy in terms of onset times. Moreover, cells that initially reside in one location may migrate to another, and the whole process may include additional spatiotemporal events of cell differentiation, proliferation, and death. In contrast, the common 3D bioprinting schemes are based on rapid patterning processes in which materials and cells are positioned at their final, desired location. Although post‐printing cell differentiation, proliferation, and even migration can be induced and manipulated to some extent, the native time frame and order of events will probably not be recapitulated. The nature of these parameters, in terms of their effect on the end result of tissue formation processes, still needs to be investigated. It is clear, however, that if the course of the process, by itself, plays a substantial role in the functionality of the tissue, it will be challenging to use 3D bioprinting for regenerative medicine purposes. In any case, it is reasonable to assume that there are variables in developmental biology that are either well concealed or too complicated to be recapitulated or managed by current technology. Obviously, there is also no guarantee that the required know‐how will be attained in the foreseeable future. Considering the complexity of living systems, with their interwoven signal routes and numerous feedback loops, it may not be unrealistic to consider a situation in which biology will eventually put a glass ceiling above our heads. While this may considerably hinder progress toward clinical application, it should be remembered that 3D bioprinting is a means, not an end. That is to say that if regenerative medicine is an ultimate goal, maybe fabrication of functional substitutions for malfunctioning tissues and organs will eventually be realized via alternative technologies.

The third scenario depicts a situation in which technologies other than 3D bioprinting will eventually dominate TE, or at least some of its derived clinical developments and applications. For instance, let us assume that highly functional, 3D bioprinted complex tissues and even organs can be fabricated, but only by a process that requires an enormous amount of resources, making them inaccessible to healthcare providers. For example, we mentioned the huge number of cells required for the construction of engineered human organs. While reaching these numbers may not be a completely uncrossable barrier, it may require an exceptionally prolonged and costly process in the absence of much improved culturing technologies. Another example in this regard is the recapitulation of the fine architectures that characterize living tissues. As discussed, the rapid advances in fabrication techniques endow researchers with the capacity to generate complicated geometries at very high resolution. These techniques, however, suffer from a low throughput and compositional complexity. Thus, scientists largely rely on spontaneous cell‐organization processes to create, for instance, the finest capillary networks in small, engineered cellular constructs. Indeed, such processes may take place when providing cells with a rough spatial guidance and proper biochemical cues. It is also known that such processes rapidly and efficiently occur as part of the natural response to tissue damage.^[^ ^25^ ^]^ We cannot be sure, however, that these processes will suffice to establish a proper blood vessel infrastructure that is capable of supporting full‐size, engineered, functional organs. And, in case they do not, ultra‐high resolution printing procedures, which will probably be adapted in the future for higher compositional complexity, may be the only available solution.^[^ ^24^ ^]^ Nevertheless, the cost of massive use of these techniques, required for generating full‐scale organs for transplantation, may make the process practically unattainable for most patients.

Thus, if top‐notch, state‐of‐the art 3D bioprinting technology does not yield affordable, transplantation‐ready engineered body parts, what solution will modern medicine offer to patients with failing tissues and organs? If artificial means for mimicking or bypassing developmental processes are not the answer, natural developmental processes may be harnessed for this purpose. While still immature and ethically controversial, somatic cell nuclear transfer techniques enable the generation of a genetic clone of an adult animal.^[^ ^93^, ^94^ ^]^ It may be possible that in the future, this technology will allow scientists to initiate developmental processes that yield functional organs without the necessity of generating a conscious, living, whole organism. Another intriguing option is to use animals as a source of transplantable tissues and organs (xenotransplantation), with recent interesting research performed on genetically modified pigs.^[^ ^95^ ^]^ An entirely different direction may be the construction of artificial, synthetic organs.^[^ ^96^, ^97^, ^98^ ^]^ Although currently not sufficiently developed to provide fully functional implantable or wearable replacements for malfunctioning organs, the technology may reach that point in the future.

With that being said, we believe that 3D‐bioprinting of functional tissues and organs will continue to develop, even in the case where it is not the method of choice for manufacturing body part substitutes. This is because research may substantially benefit from the ability to control the structure and composition of these native‐like structures. For instance, 3D bioprinting may enable the incorporation of genetically modified cells expressing a reporter gene at specific locations in an engineered organ. It may also allow the integration of actuators and sensors that will shed light on hard‐to‐detect physiological processes and activities. This freedom of design, not offered by the described alternatives, may rise above the cost of production, maintaining the demand for these functional printed bioconstructs.

## 4 Conclusions

3D printing is an ingenious concept and a groundbreaking technology that impacts a wide range of disciplines such as architecture, industrial design, manufacturing, and art. Owing to its power to grant users the exceptional capability to quickly and accurately convert a digital design into a 3D physical object, 3D printing gradually caught the attention of tissue engineers. Today, 3D bioprinting is one of the most desirable tools in TE, offering an advanced means (and in many cases the only means) for the construction of complex biostructures. Major efforts are now being made to refine the process, aiming to improve printing accuracy and speed as well as the complexity of the resulting printout. Indeed, the latest works overviewed in this article prove that motivation and creativity can be combined with knowledge and talent to achieve these goals. Aided by knowledge in cell biology and the expected advances in our understanding of developmental processes, 3D bioprinting may be the spearhead in the future of TE, taking it to new and higher levels. Obviously, any major advances in this field will open new gates, expedite developments, and accelerate progress in applicative regenerative medicine. Will 3D bioprinting be the technology of choice for generating transplantation‐ready, complex engineered tissues and organs? Or, should we humbly ask, will any technology bring us to this point in the foreseeable future? We believe that biology holds the key. It may or may not comply with attempts to control and manipulate it according to our needs and desires. But while we wonder if and when the transplantation of complex biofabricated constructs will become a routine clinical procedure, it seems that 3D bioprinting technology is rapidly evolving toward the realization of this vision.

## Conflict of Interest

The authors declare no conflict of interest.
